# Pericoronary, but Not Epicardial, Cardiac Fat Thickness Is Associated with Sarcopenia in Hospitalized Older Adults

**DOI:** 10.3390/medicina62061115

**Published:** 2026-06-08

**Authors:** Kübra Erdoğan, Rıdvan Erten, Rana Tuna Doğrul, Velihan Çayhan, Serap Boz, İmran Ceren, Ertuğrul Demirel, Hande Selvi Öztorun, Güneş Eken, Kamile Sılay

**Affiliations:** 1 Division of Geriatrics, Department of Internal Medicine, Ankara Bilkent City Hospital, 06200 Ankara, Türkiye; dr.ridvanerten@gmail.com (R.E.); rana_tuna@hotmail.com (R.T.D.); serapsen85@hotmail.com (S.B.); drertugruldemirel@gmail.com (E.D.); 2 Department of Radiology, Ankara Bilkent City Hospital, 06200 Ankara, Türkiye; cayhanvelihan@gmail.com; 3 Department of Cardiology, Dr. Abdurrahman Yurtaslan Ankara Oncology Training and Research Hospital, 06200 Ankara, Türkiye; drimranceren@gmail.com; 4 Division of Geriatrics, Department of Internal Medicine, Faculty of Medicine, Ankara Yıldırım Beyazıt University, 06010 Ankara, Türkiye; drhandeslv@hotmail.com (H.S.Ö.); kamilesilay@hotmail.com (K.S.); 5 Division of Geriatrics, Department of Internal Medicine, Faculty of Medicine, Health Science University, 06200 Ankara, Türkiye; guneseken@yahoo.com

**Keywords:** sarcopenia, body composition, pericoronary adipose tissue, aged

## Abstract

*Background and Objectives*: Sarcopenia is a major geriatric condition associated with functional decline and adverse outcomes. Cardiac fat depots exhibit metabolic activity and are linked to cardiometabolic risk; however, the extent to which epicardial adipose tissue (EAT) and pericoronary adipose tissue (PCAT) quantified on standard thoracic computed tomography (CT) scans are associated with sarcopenia in older inpatients remains inadequately explored. This study investigated the associations of EAT and PCAT thickness with sarcopenia. *Materials and Methods*: This is a retrospective observational study using CT data obtained for clinical purposes and routine geriatric assessment data. In this single-center retrospective study, 101 inpatients aged ≥65 years who underwent unenhanced thoracic CT and bioelectrical impedance analysis within 3 months were included. EAT and PCAT thicknesses were measured using standardized methods. Sarcopenia status was based on a previously established clinical diagnosis according to EWGSOP2 criteria. Multivariable logistic regression, body mass index (BMI)-stratified analyses, and ROC curve evaluations were performed. *Results*: The participants had a mean age of 78.5 ± 7.6 years; 54.5% were female. Fifty-five patients (54.5%) met the diagnostic criteria for sarcopenia. PCAT was significantly thicker in sarcopenic participants relative to non-sarcopenic ones (12.25 ± 2.50 vs. 11.17 ± 2.32 mm, *p* = 0.028), while no corresponding difference was observed for EAT (*p* = 0.959). After controlling for age, sex, and BMI, each 1 mm increase in PCAT thickness was independently associated with an increased odds of sarcopenia (OR 1.399, 95% CI 1.133–1.727, *p* = 0.002). With the addition of the PCAT, the discriminatory power was modest (AUC 0.637 overall and 0.715 for BMI ≥25 kg/m^2^). In exploratory stratified analyses, the association was numerically larger in the BMI ≥25 kg/m^2^ subgroup (OR 1.48, *p* = 0.008); however, the formal BMI-by-PCAT interaction was not statistically significant (*p* = 0.290), so this subgroup finding is considered hypothesis-generating. *Conclusions*: PCAT thickness measured on routine non-contrast thoracic CT was associated with prevalent sarcopenia, whereas EAT thickness was not. ROC analyses showed modest discrimination; therefore, any proposed cut-offs should be considered exploratory and require prospective validation and external confirmation before clinical use.

## 1. Introduction

Sarcopenia is a progressive skeletal muscle condition whose prevalence increases steadily with age; it is characterized by declining muscle strength and decreasing muscle mass, with secondary effects on mobility, self-sufficiency, and survival [1,2].

Among hospitalized older individuals, extended immobilization, systemic inflammation, and inadequate nutritional intake often accelerate this process [3]. Early recognition of sarcopenia is therefore a key component of thorough geriatric evaluation. Adipose tissue actively contributes to chronic low-grade inflammation and may create a biological milieu conducive to the development of sarcopenia. Within cardiac fat compartments, both epicardial and pericoronary deposits have attracted substantial attention due to their association with cardiovascular disease [4]. Alterations in lipid metabolism, such as cholesteryl ester transfer protein (CETP), have been associated with an increased risk of cardiometabolic disease, providing additional context for the systemic metabolic environment in which regional adiposity and sarcopenia may coexist [5]. Previous research showing an inverse relationship between epicardial fat accumulation and handgrip strength implies that cardiac fat load may mirror age-dependent loss of musculoskeletal integrity [6,7]. Moreover, sarcopenia and cardiovascular disease appear to influence each other bidirectionally, and their co-occurrence may worsen disease severity in settings such as coronary atherosclerosis and heart failure [8].

Routine thoracic CT offers a practical opportunity to evaluate cardiac fat on images obtained for standard clinical purposes. Existing evidence has connected CT-derived EAT volume and density to coronary atherosclerosis and unfavorable cardiovascular outcomes [9]. However, cardiac fat likely does not behave as a uniform compartment. Because of its immediate anatomic proximity to the coronary arterial wall, PCAT is considered to more accurately reflect localized inflammatory and cardiometabolic signaling than the epicardial fat depot as a whole [10].

In this study, we prioritized linear thickness measurements of PCAT on routine, unenhanced thoracic CT to emphasize the feasibility and ‘opportunistic’ utility of this approach in geriatric clinical practice. While volumetric or attenuation-based analyses are often preferred in dedicated cardiac research, they typically require specialized software and ECG-gated protocols that are not part of standard care for most hospitalized older adults. By utilizing linear measurements on non-contrast scans which are frequently performed for diverse clinical indications in this population we aim to demonstrate a pragmatic method for integrating cardiometabolic biomarkers into multidimensional geriatric assessment without additional radiation exposure or specialized post-processing requirements.

Recent advances in opportunistic imaging have underscored the potential of routinely acquired CT scans to capture multidimensional biomarkers of biological aging. Beyond conventional cardiovascular risk assessment, imaging-derived metrics of adipose distribution may reflect systemic metabolic dysregulation and age-related decline in musculoskeletal resilience [11]. In this framework, cardiac adipose tissue particularly regionally distinct depots such as PCAT may serve as an integrative imaging phenotype that links cardiometabolic burden to sarcopenic vulnerability. Elucidating this relationship may therefore provide mechanistic insight into the shared pathways underlying cardiovascular and musculoskeletal aging, with potential implications for risk stratification and personalized management in geriatric populations.

Notwithstanding this rationale, simultaneous evaluation of EAT and PCAT in relation to sarcopenia in geriatric inpatients remains limited. Therefore, we conducted a retrospective observational study to examine the association between CT-derived EAT/PCAT thickness and sarcopenia defined using EWGSOP2 components from routine comprehensive geriatric assessment. We also explored the discriminative performance of these measures within clinical models, without aiming to establish a definitive diagnostic threshold.

## 2. Materials and Methods

### 2.1. Study Population

We performed a retrospective observational study at a single institution, recruiting 101 patients aged 65 or older who were hospitalized on a geriatric ward during June 2024 to January 2025. Inclusion required a completed comprehensive geriatric assessment together with thoracic CT and bioelectrical impedance analysis performed within a 3-month interval, irrespective of whether bioelectrical impedance analysis (BIA) preceded or followed CT. Every thoracic CT examination was clinically indicated rather than research-driven. Patients presenting with acute infections, active malignancies, or active systemic rheumatic conditions were not included. Approval was obtained from the Ethics Committee for Clinical Research (TABED 1/889/2025), and all procedures conformed to the principles outlined in the Declaration of Helsinki.

Age, sex, with whom the patient lived, educational status, smoking and alcohol use, body mass index (BMI), accompanying diseases, incontinence, falls, pressure ulcer, number of drugs used, and laboratory findings were recorded.

### 2.2. Comprehensive Geriatric Assessment

As part of the comprehensive geriatric assessment, patients’ functional status was evaluated using the Katz Activities of Daily Living Scale (ADL) [12] and the Lawton–Brody Instrumental Activities of Daily Living Scale (IADL) [13]. Depression was screened with the short form of the Yesavage Geriatric Depression Scale (GDS) [14]. At the same time, the risk of malnutrition was assessed using the short form of the Mini Nutritional Assessment (MNA-SF) [15]. To evaluate cognitive status, medical histories were obtained from both patients and their caregivers, and the Mini-Mental State Examination (MMSE) [16] was administered as one screening tool.

### 2.3. Chest Computed Tomography

Each thoracic CT examination was performed with the patient in the dorsal decubitus position at end-inspiration, using a high-resolution unenhanced technique with a reconstruction thickness of 1–1.5 mm. All images were archived in the institutional PACS, with radiation exposure adapted automatically according to patient age and body habitus. A 16-row multidetector scanner (Aquilion; Toshiba Medical Systems, Otawara, Tochigi, Japan) was used, with scan coverage extending from the thoracic inlet to the costophrenic recesses. The tube voltage was set to 100 kVp, with automatic modulation of tube current across a range of 20–400 mA and reconstruction intervals of 1–1.5 mm. A senior radiologist reviewed all datasets.

**Figure 1:**
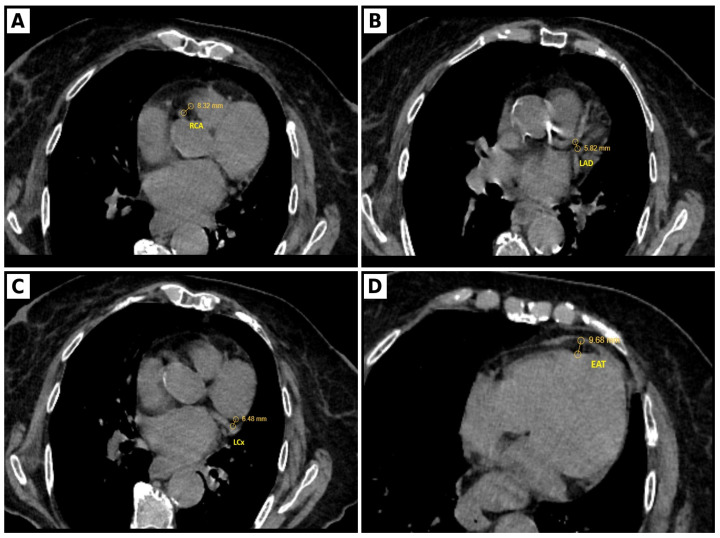
Representative non-contrast thoracic CT images illustrating the measurement technique for pericoronary adipose tissue (PCAT) and epicardial adipose tissue (EAT). PCAT was measured as the perpendicular linear thickness of the adipose layer surrounding each proximal coronary artery: (**A**) right coronary artery (RCA) at the right atrioventricular groove; (**B**) left anterior descending artery (LAD) just distal to the bifurcation of the left main coronary artery; (**C**) left circumflex artery (LCx) at the left atrioventricular groove. The arithmetic mean of the three territorial measurements constituted the composite PCAT value. (**D**) EAT was measured as the perpendicular thickness of the adipose layer between the outer myocardial boundary and the visceral pericardium, anterior to the right ventricular free wall. Images were reconstructed at 1–1.5 mm slice thickness, with window settings optimized to delineate fat boundaries. The measurements shown are from a single representative patient and are intended to illustrate the anatomical landmarks and measurement technique; the displayed EAT value is the raw measurement before application of the 1 mm correction factor described in the Methods. Calipers and values (in millimetres) are those generated on the PACS workstation.

All thoracic CT measurements were performed by a single senior radiologist using a standardised protocol. PCAT thickness was measured on 1–1.5 mm thin slice reconstructions as the mean of linear measurements across the RCA, LAD, and LCx territories; EAT was measured as the maximal perpendicular thickness anterior to the right ventricular free wall. Oblique or nonperpendicular sections were excluded to limit motion-related error in these non-ECG gated examinations. To assess measurement robustness, the reader remeasured a stratified random subset of 30 scans after an interval of at least two weeks, blinded to the initial values; the intraobserver intraclass correlation coefficients (ICC [2,1], two-way random-effects, absolute agreement) were 0.968 (95% CI 0.93–0.98) for PCAT and 0.965 (95% CI 0.93–0.98) for EAT. Representative measurements illustrating the anatomical landmarks for each coronary territory and for EAT are shown in Figure 1.

Before performing measurements, the display window settings were optimized to delineate fat boundaries and the pericardial outline clearly. EAT was defined for measurement purposes as the adipose layer interposed between the outer myocardial boundary and the visceral pericardium. EAT thickness was quantified on basal short axis reformats at the level of the right ventricular free wall, according to a previously reported and validated technique [17,18].

Thickness was sampled perpendicular to the cardiac surface at three predefined locations along the right ventricular free wall, roughly corresponding to the 25%, 50%, and 75% reference points; the mean of these three readings was used in statistical analyses. A standard 1 mm correction factor was subtracted to reduce measurement error attributable to bordering vascular structures, and non-perpendicular (oblique) measurements were systematically excluded. The 1 mm correction applies only to EAT and not to PCAT.

PCAT corresponded to the adipose layer that envelops the major epicardial coronary vessels. Measurements were performed separately for each of the three coronary territories: RCA, LAD, and LCx. At each site, the maximum perpendicular distance from the myocardial surface to the visceral pericardial boundary was documented, and the arithmetic average of these three readings constituted the composite PCAT value.

### 2.4. Muscle Assessment

Body weight and standing height were obtained in light clothing and without footwear. Waist girth was taken at a horizontal plane midway between the costal margin and the iliac crest, and hip girth at the level of greatest gluteal projection.

A Bodystat Quadscan 4000 device (Bodystat, Sulby, UK) was used to assess body composition using bioelectrical impedance. Patients lay supine, metallic accessories were removed, and standardized preparatory conditions—including an overnight fast and bladder emptying were observed. Electrode pairs were attached to the dorsal surfaces of the ipsilateral hand and foot at both distal and proximal sites. BIA was not performed in patients with cardiac pacemakers or other implanted electronic devices, those exhibiting clinically relevant peripheral edema, or those with pronounced electrolyte imbalances.

The phase angle, reflecting the reactance-to-resistance ratio, served as a proxy for cellular membrane quality. Total skeletal muscle mass was derived from whole-body bioelectrical impedance data using the prediction equation described by Janssen et al.: SMM (kg) = (stature^2^/resistance × 0.401) + (sex [male = 1, female = 0] × 3.825) + (age × −0.071) + 5.102 [19]. SMMI was subsequently obtained by normalizing SMM to the square of height. Muscle mass was classified as low at SMMI values below 9.2 kg/m^2^ in men and below 7.4 kg/m^2^ in women, following the Turkish-population reference values proposed by Bahat et al. [20].

A Takei 5101 digital dynamometer (TKK) was used to quantify maximum grip force. The participant sat with the dominant forearm flexed to 90°, and three maximal contractions were performed; the highest reading was retained. Grip weakness was defined as a peak value below 32 kg in men and below 22 kg in women, using the Turkish-population reference cutoffs of Bahat et al. [20]. Lower-limb performance was evaluated with a five-cycle sit-to-stand test on a standard-height chair; participants kept both arms held against the chest throughout. The median of three trial durations was recorded, and values of 15 s or above were classified as impaired.

Habitual walking pace was assessed by timing a 4 m level course at the participant’s self-chosen pace; a result slower than 0.8 m/s signified impaired physical functioning. Sarcopenia status had been established clinically according to the EWGSOP2 algorithm [1], applied with Turkish-population reference cutoffs from Bahat et al. [20]. Under EWGSOP2, isolated weakness indicates probable sarcopenia, combined low strength and reduced muscle mass yield confirmed sarcopenia, and the additional finding of reduced walking speed defines severe sarcopenia [1]. BIA-derived SMMI cut-points were used to characterize low muscle mass in accordance with these criteria. Muscle mass (BIA-derived SMMI) was available in all 101 patients. Handgrip dynamometry yielded numeric values in 89 of 101 patients (88.1%); in the remaining 12 patients (11.9%), grip dynamometry could not be performed because of severe cognitive impairment, ICU-acquired weakness, or hand pathology, and these patients were operationally classified as having low strength in the retrospective algorithm. This approach reflects clinically meaningful inability to complete strength assessment but may introduce outcome misclassification; concordance and sensitivity analyses were therefore performed and are reported in the Supplementary Materials. The five-times sit-to-stand test (n = 44) served as a corroborative strength measure. Physical performance (4 m gait speed, SPPB) was used for severity grading when available but did not affect the binary sarcopenia outcome used in this study.

### 2.5. Statistical Analysis

Statistical computations were primarily conducted with SPSS 25.0 (IBM Corporation, Armonk, NY, USA). Further calculations, namely ROC curve comparisons and BMI subgroup models, were performed in Python 3.12 using the scipy and statsmodels libraries. A two-sided *p*-value threshold of <0.05 defined statistical significance.

The Kolmogorov–Smirnov test (whole sample, n > 50) and the Shapiro–Wilk test (smaller subsets) were applied to verify the normality of continuous variables. These tests were complemented by evaluation of skewness, kurtosis, variation coefficients, histograms, and Q–Q plots. Continuous data conforming to a Gaussian distribution are stated as mean ± SD; those that did not are stated as median (IQR). Categorical data are displayed as counts with percentages.

Normally distributed continuous variables were compared between sarcopenic and non-sarcopenic groups using the Student *t*-test; the Mann–Whitney U test was used when data failed normality assumptions. Categorical differences were evaluated by the χ^2^ test, or by Fisher’s exact method if any expected cell count was below five. Pearson or Spearman correlation coefficients selected based on variable distributions quantified the relationship of fat thickness with muscle indices, and all correlations were recalculated within each sex.

The cardiac fat–sarcopenia relationship was modelled using sequential binary logistic regression across three model-building steps: the first included fat thickness alone, the second added age and sex, and the third included BMI. Results are reported as OR (95% CI). Model calibration was assessed using the Hosmer–Lemeshow statistic, and explained variance was assessed using Nagelkerke R^2^.

To explore whether the PCAT–sarcopenia association differed by adiposity status, the cohort was dichotomized at BMI 25 kg/m^2^, and separate age- and sex-adjusted logistic models were constructed within each BMI stratum. A multiplicative interaction term (BMI stratum × PCAT thickness) was added to the full model and formally tested. Within BMI strata, the relationship between PCAT thickness and skeletal muscle mass was assessed using Spearman correlation. Variance inflation factors were examined to assess collinearity. The linearity of the logit assumption was evaluated using the Box–Tidwell test and by examining quartile-specific log-odds. The potential for a non-linear relationship was assessed by adding a quadratic term (PCAT^2^) to the regression models. Cook’s distance was computed to identify influential observations, and sensitivity analyses were performed excluding these cases. The adequacy of sample size relative to the number of predictors was assessed using the events-per-variable (EPV) criterion.

ROC curves were plotted to assess how effectively PCAT thickness discriminated sarcopenia [21]; the AUC was estimated, and the optimal threshold was determined using the Youden J index. Sensitivity, specificity, PPV, and NPV were subsequently calculated for the whole cohort and for each BMI subgroup.

Given that the central hypothesis was prespecified, no multiplicity correction was applied to the primary analysis; all subgroup examinations were considered exploratory. Of 117 eligible records reviewed, 101 had complete EAT and PCAT measurements and therefore constituted the primary analytic cohort; 16 records were excluded because CT-derived fat thickness values were unavailable. Within the final analytic cohort, missing data for variables included in the regression models (EAT, PCAT, SMM, SMMI, age, sex, BMI) were negligible. Among secondary variables used in comprehensive geriatric assessment (Katz, Lawton, MNA, and GDS), missing data ranged from 28% to 49% and were handled using complete-case analysis for each analysis. The availability and operational handling of EWGSOP2 muscle strength components (handgrip and 5 × sit-to-stand) are described in Section 2.4. Missing data were confined to secondary comprehensive geriatric assessment instruments and did not affect the sarcopenia classification or any variable used in the primary regression models; all components required for the EWGSOP2 outcome, together with PCAT and EAT, were available for the full analytic cohort (n = 101).

As diabetes mellitus differed between groups at baseline, an additional sensitivity model (Model E) further adjusted for diabetes; the events per variable ratio for this model was 11.0.

## 3. Results

### 3.1. Characteristics of the Study Cohort

One hundred and one hospitalized older patients with complete EAT and PCAT data formed the final study population. These 101 patients were drawn from 117 eligible records, of which 16 were excluded because CT-derived fat thickness measurements were unavailable. The sample comprised 55 women (54.5%) and 46 men (45.5%), with an average age of 78.5 ± 7.6 years. A total of 55 patients (54.5%) received a sarcopenia diagnosis, while the other 46 (45.5%) did not satisfy the diagnostic requirements (Table 1). The median interval between thoracic CT and BIA was 36 days (IQR 10–81 days; range 0–90 days); 46 patients (45.5%) were assessed within 30 days, 58 (57.4%) within 60 days, and all 101 (100%) within 90 days, in accordance with the inclusion criterion. The interval did not differ between sarcopenic and non-sarcopenic groups (*p* = 0.635). Regarding the EWGSOP2 components, BIA-derived SMMI was available for all 101 patients and was below the reference cut-off in 55; numeric handgrip values were available in 89 patients and were below the cut-off in 67, while the 12 patients without a measurable grip were operationally classified as having low strength, giving 79 patients with low strength overall. A physical-performance measure (gait speed or five-times sit-to-stand) was available in 52 of the 53 patients with combined low strength and low muscle mass. An internal consistency audit re-applying the formal EWGSOP2 algorithm reproduced the recorded clinical sarcopenia label in 99 of 101 patients (98.0%; Cohen’s κ = 0.960); the component breakdown is shown in Supplementary Figure S1.

**Table 1:** Baseline characteristics of the study population, stratified by sarcopenia status.

Variable	n Available	Total (n = 101)	No Sarcopenia (n = 46)	Sarcopenia (n = 55)
Demographics				
Age, years	101	78.5 ± 7.6	77.6 ± 7.5	79.2 ± 7.7
Female, n (%)	101	55 (54.5)	26 (56.5)	29 (52.7)
BMI, kg/m^2^	101	25.8 (22.9–29.4)	27.1 (23.5–32.8)	25.3 (21.5–27.7)
Weight, kg	101	69.1 ± 15.3	72.4 ± 15.2	66.3 ± 14.9
Height, cm	101	161.1 ± 10.2	160.0 ± 9.3	162.0 ± 10.9
Education ≤ primary, n (%)	100	70 (70.0)	34 (75.6)	36 (65.5)
Comorbidities				
Hypertension, n (%)	101	67 (66.3)	32 (69.6)	35 (63.6)
Diabetes mellitus, n (%)	101	45 (44.6)	27 (58.7)	18 (32.7)
Coronary artery disease, n (%)	101	37 (36.6)	18 (39.1)	19 (34.5)
Heart failure, n (%)	72	14 (19.4)	7 (24.1)	7 (16.3)
Atrial fibrillation, n (%)	72	14 (19.4)	5 (17.2)	9 (20.9)
Stroke, n (%)	72	14 (19.4)	8 (27.6)	6 (14.0)
COPD, n (%)	72	6 (8.3)	1 (3.4)	5 (11.6)
Dementia, n (%)	71	18 (25.4)	5 (17.9)	13 (30.2)
Chronic kidney disease, n (%)	72	13 (18.1)	5 (17.2)	8 (18.6)
Cancer, n (%)	72	8 (11.1)	1 (3.4)	7 (16.3)
Depression, n (%)	70	21 (30.0)	8 (27.6)	13 (31.7)
Geriatric Assessment				
Katz ADL (0–6)	72	4.0 (1.0–5.0)	3.0 (1.0–5.0)	4.0 (1.5–5.0)
Lawton IADL (0–8)	72	3.0 (0.0–6.2)	3.0 (0.0–7.0)	3.0 (0.0–6.0)
MMSE (0–30)	55	24.0 (20.5–27.0)	25.0 (21.5–27.0)	23.0 (20.0–27.2)
MNA-SF (0–14)	68	9.0 (5.0–11.0)	9.0 (4.5–12.0)	9.0 (5.0–10.0)
GDS (0–15)	52	5.0 (3.0–9.0)	6.0 (2.0–9.2)	4.5 (3.0–8.0)
Clinical frailty	71	6.0 (4.0–7.0)	6.0 (4.0–7.0)	5.5 (4.0–7.0)
Number of medications	72	7.0 (4.0–9.0)	7.0 (5.0–10.0)	6.0 (4.0–9.0)
Geriatric Syndromes				
Polypharmacy, n (%)	72	52 (72.2)	20 (69.0)	32 (74.4)
Falls, n (%)	71	33 (46.5)	16 (57.1)	17 (39.5)
Urinary incontinence, n (%)	70	46 (65.7)	18 (64.3)	28 (66.7)
Pressure ulcer, n (%)	69	9 (13.0)	6 (21.4)	3 (7.3)
Malnutrition, n (%)	66	29 (43.9)	11 (39.3)	18 (47.4)
Dysphagia, n (%)	69	19 (27.5)	8 (29.6)	11 (26.2)
Constipation, n (%)	70	23 (32.9)	11 (40.7)	12 (27.9)
Sleep disorder, n (%)	70	28 (40.0)	11 (39.3)	17 (40.5)
Chronic pain, n (%)	70	23 (32.9)	10 (37.0)	13 (30.2)

Footnote: Data are presented as mean ± SD or median (IQR) according to distribution, or as n (%). The “n available” column shows the number of patients with available data for each variable; percentages were calculated using this denominator. The analytic dataset (EAT, PCAT, SMM, SMMI, age, sex, BMI, and the sarcopenia outcome) was complete for all 101 patients; variable-specific missingness applied only to descriptive secondary variables. *p* values from Student’s *t*-test, Mann–Whitney U, χ^2^, or Fisher’s exact test as appropriate. ADL, activities of daily living; BMI, body mass index; COPD, chronic obstructive pulmonary disease; GDS, Geriatric Depression Scale; IADL, instrumental activities of daily living; MMSE, Mini-Mental State Examination; MNA-SF, Mini Nutritional Assessment–Short Form.

Data are presented as mean ± SD or median (IQR) according to distribution. *p* values from Student’s *t*-test, Mann–Whitney U, χ^2^, or Fisher’s exact test as appropriate. Percentages are calculated using the number of patients with available data for each variable, which varied because of variable-specific missingness. Data were complete (n = 101) for age, sex, BMI, weight, height, education, hypertension, diabetes, and coronary artery disease. For the remaining comorbidities (heart failure, atrial fibrillation, COPD, stroke, chronic kidney disease, cancer) data were available for 72 patients, and for dementia and depression for 71 and 70 patients, respectively. Among the geriatric assessment instruments, data were available for Katz and Lawton in 72 patients, MNA-SF in 68, MMSE in 55, the Geriatric Depression Scale in 52, and the Clinical Frailty Scale in 71. These secondary variables were used for descriptive purposes only and were not entered into the primary regression models.

### 3.2. Comparison by Sarcopenia Status

**Figure 2:**
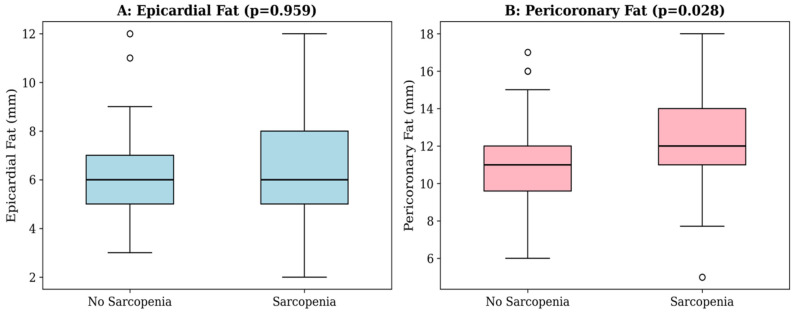
EAT and PCAT thickness by sarcopenia status. (**A**) EAT thickness did not differ between sarcopenic and non-sarcopenic participants (Mann–Whitney U test, *p* = 0.959). (**B**) PCAT thickness was elevated in sarcopenic participants (Student’s *t*-test, *p* = 0.028).

PCAT averaged 11.76 ± 2.47 mm, and EAT had a median of 6.0 mm (IQR 5.0–8.0). The mean skeletal muscle mass across the cohort was 21.82 ± 6.08 kg, and the median SMMI was 8.2 kg/m^2^ (IQR 7.0–9.3). PCAT thickness was greater in sarcopenic patients than in their non-sarcopenic counterparts (12.25 ± 2.50 mm vs. 11.17 ± 2.32 mm, *p* = 0.028). EAT thickness, by contrast, did not differ meaningfully between the two groups (6.35 ± 2.20 mm vs. 6.31 ± 1.94 mm, *p* = 0.959). As anticipated, both skeletal muscle mass and SMMI were substantially lower among sarcopenic participants (18.97 ± 4.95 kg vs. 25.24 ± 5.56 kg and 7.11 ± 1.18 kg/m^2^ vs. 9.82 ± 1.98 kg/m^2^, respectively; both *p* < 0.001) (Table 2) (Figure 2).

**Table 2:** Descriptive characteristics of body composition and cardiac fat measurements.

Variable	Total (n: 101)	No Sarcopenia (n: 46)	Sarcopenia (n: 55)	*p* Value
Epicardial fat thickness (mm)	6.0 (5.0–8.0)	6.0 (5.0–7.0)	6.0 (5.0–8.0)	0.959
Pericoronary fat thickness (mm)	11.76 ± 2.47	11.17 ± 2.32	12.25 ± 2.50	**0.028**
Skeletal muscle mass (kg)	21.82 ± 6.08	25.24 ± 5.56	18.97 ± 4.95	**<0.001**
Skeletal muscle mass index (kg/m^2^)	8.2 (7.0–9.3)	9.5 (8.7–10.4)	7.0 (6.3–8.1)	**<0.001**
Body mass index (kg/m^2^)	25.8 (22.9–29.4)	27.1 (23.5–32.8)	25.3 (21.5–27.7)	**0.007**

Continuous variables are presented as mean ± SD or median (IQR), depending on the results of the normality assessment. Comparisons were performed using Student’s *t*-test (PCAT, SMM) or Mann–Whitney U test (EAT, SMMI, BMI). Bold *p*-values indicate statistical significance (*p* < 0.050).

### 3.3. Adipose Tissue-Muscle Correlations

**Figure 3:**
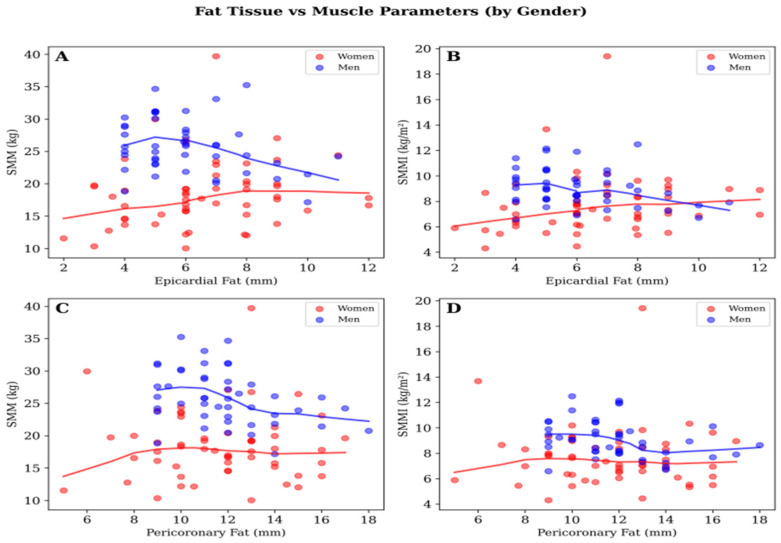
Sex-stratified scatter plots of cardiac fat thickness versus muscle parameters. Women are shown in red and men in blue; LOWESS curves illustrate potential non-linear trends. Spearman correlation coefficients are reported in the text. (**A**) Epicardial fat thickness vs skeletal muscle mass (SMM). (**B**) Epicardial fat thickness vs skeletal muscle mass index (SMMI). (**C**) Pericoronary fat thickness vs skeletal muscle mass (SMM). (**D**) Pericoronary fat thickness vs skeletal muscle mass index (SMMI).

Across the full cohort, neither EAT nor PCAT thickness showed a statistically significant correlation with skeletal muscle mass. When the analysis was stratified by sex, however, a distinct pattern emerged among men: higher PCAT thickness was associated with lower skeletal muscle mass (Spearman rho = −0.380, *p* = 0.009). No analogous relationship was detected in women (Figure 3). PCAT thickness was positively correlated with BMI across the full cohort (Spearman ρ = 0.34, *p* < 0.001).

### 3.4. Main Analysis: Hierarchical Regression Models

Hierarchical logistic regression revealed a consistent association between PCAT thickness and sarcopenia. In the unadjusted model, each 1 mm increase in PCAT thickness was associated with a higher odds of sarcopenia (OR = 1.207, 95% CI 1.016–1.433; *p* = 0.032).

**Figure 4:**
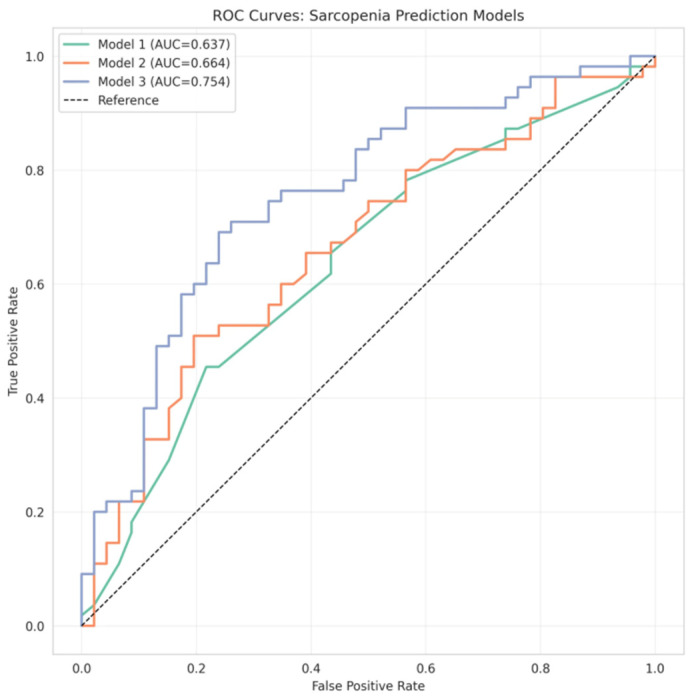
Receiver operating characteristic curves for logistic regression models for discrimination of sarcopenia. The model incorporating age, sex, and BMI achieved the greatest discriminative performance (AUC = 0.754).

This association remained after controlling for age and sex (OR = 1.212, 95% CI 1.019–1.443; *p* = 0.030) and strengthened further when BMI was added (OR = 1.399, 95% CI 1.133–1.727; *p* = 0.002). EAT thickness was not significantly associated with sarcopenia in any of the models. Discriminative capacity improved progressively with the inclusion of covariates, and the model incorporating age, sex, and BMI achieved the highest AUC (0.754) (Table 3; Figure 4). All models demonstrated adequate calibration according to the Hosmer–Lemeshow test (*p* > 0.38). The Box–Tidwell test confirmed that the logit linearity assumption was satisfied for PCAT thickness (*p* = 0.82). Variance inflation factors were below 1.15 for all predictors, indicating no multicollinearity. The events-per-variable ratio was adequate for Models A through C (EPV ≥ 11.2). The fully adjusted model, including comorbidities (Model D), yielded an EPV of 6.4, falling below the recommended threshold of 10; its results are therefore presented as a sensitivity analysis rather than a primary finding and should be interpreted accordingly. Cook’s distance identified seven influential observations; a sensitivity analysis excluding these cases yielded a larger effect estimate (OR = 1.952, *p* < 0.001), suggesting that the primary analysis provides a conservative estimate of the association. In a diabetes adjusted sensitivity model (Model E: age, sex, BMI, diabetes), the association was essentially unchanged (adjusted OR 1.39, 95% CI 1.12–1.71, *p* = 0.003); diabetes was not an independent predictor (OR 0.47, 95% CI 0.18–1.18, *p* = 0.107), and the likelihood ratio test versus the primary model was nonsignificant (chi-square = 2.61, *p* = 0.106).

**Table 3:** Hierarchical Logistic Regression for PCAT and Sarcopenia.

Model	OR (95% CI)	*p* Value
Model 1 (crude)	1.21 (1.02–1.43)	**0.032**
Model 2 (adjusted for age and sex)	1.21 (1.02–1.44)	**0.030**
Model 3 (additionally adjusted for BMI)	1.40 (1.13–1.73)	**0.002**

Bold *p*-values indicate statistical significance (*p* < 0.050).

### 3.5. BMI-Stratified Findings

When stratified by BMI, those with BMI ≥ 25 kg/m^2^ exhibited thicker PCAT than those with BMI < 25 kg/m^2^ (12.38 ± 2.30 mm vs. 10.88 ± 2.46 mm, *p* = 0.002). Nevertheless, sarcopenia prevalence was higher in the lower BMI stratum (64.3% vs. 47.5%). In logistic models adjusted for age and sex, the PCAT–sarcopenia association remained significant among participants with BMI ≥ 25 kg/m^2^ (OR = 1.48, 95% CI 1.11–1.98; *p* = 0.008), but was not significant in the BMI < 25 kg/m^2^ subgroup (OR = 1.20, 95% CI 0.90–1.60; *p* = 0.207) (Supplementary Figures S2 and S3; Table 4). However, the formal multiplicative interaction between BMI stratum and PCAT thickness was not statistically significant (*p* for interaction = 0.290), and these subgroup findings should therefore be interpreted as exploratory.

**Table 4:** BMI-stratified association of PCAT with sarcopenia and skeletal muscle mass.

BMI Category	PCAT Thickness (mm), Mean ± SD	Sarcopenia Prevalence (%)	OR for Sarcopenia (95% CI) *	*p* Value	Correlation with Skeletal Muscle Mass (Rho)	*p* Value
BMI < 25 kg/m^2^	10.88 ± 2.46	64.3	1.20 (0.90–1.60)	0.207	0.086	0.590
BMI ≥ 25 kg/m^2^	12.38 ± 2.30	47.5	1.48 (1.11–1.98)	**0.008**	−0.321	**0.013**
Overall	11.76 ± 2.47	54.5	1.21 (1.02–1.44)	**0.030**	−0.101	0.313

* Age- and sex-adjusted logistic regression. Correlation coefficients are Spearman’s rho. Overall estimate corresponds to the age- and sex-adjusted model (Table 3, Model 2). Bold *p*-values indicate statistical significance (*p* < 0.050).

A comparable trend was seen in correlation analyses. Among participants with BMI ≥ 25 kg/m^2^, PCAT thickness showed an inverse correlation with skeletal muscle mass (Spearman rho = −0.321, *p* = 0.013), whereas no meaningful relationship was found in those with BMI < 25 kg/m^2^ (Spearman rho = 0.086, *p* = 0.590) (Supplementary Figure S4; Table 4).

These stratified findings are consistent with a stronger association among individuals with excess body weight. Still, they should be regarded as exploratory because the formal interaction test was not statistically significant. The algorithm reproduced the recorded clinical sarcopenia label in 99 of 101 patients (98.0%; Cohen’s κ = 0.960, indicating very high agreement). Sensitivity analyses using the algorithm-concordant subset (n = 99), an algorithm-defined outcome variable (n = 101), additional adjustment for diabetes, and CT–BIA intervals restricted to ≤60 days (n = 58) or ≤30 days (n = 46) yielded materially similar PCAT effect estimates (adjusted ORs ranging from 1.36 to 1.52; all *p* < 0.05; Supplementary Figure S5).

### 3.6. ROC Analysis and Classification Performance

ROC analysis indicated that PCAT thickness distinguished sarcopenia more accurately in the BMI ≥ 25 kg/m^2^ subgroup compared with the BMI < 25 kg/m^2^ subgroup (AUC: 0.715 vs. 0.611). These cut-offs are exploratory and are not intended as clinically actionable thresholds; external validation is required. For those with BMI ≥ 25 kg/m^2^, a threshold of 11.44 mm yielded 85.7% sensitivity, 48.4% specificity, and 78.9% negative predictive value. For the BMI < 25 kg/m^2^ subgroup, a threshold of 9.80 mm achieved 74.1% sensitivity and 46.7% specificity. For the full cohort, the AUC was 0.637 at a cut-off of 13.00 mm. Overall, these exploratory findings suggest that the association between PCAT thickness and sarcopenia may be more apparent in individuals with overweight or obesity than in leaner individuals; they do not establish any rule-in or rule-out capacity (Table 5).

**Table 5:** Classification performance of PCAT thickness for sarcopenia by BMI subgroup.

Group	n	AUC	Cutoff (mm)	Sens. (%)	Spec. (%)	PPV (%)	NPV (%)	Accuracy (%)
BMI < 25	42	0.611	9.80	74.1	46.7	71.4	50.0	64.3
BMI ≥ 25	59	0.715	11.44	85.7	48.4	60.0	78.9	66.1
All Cohort	101	0.637	13.00	45.5	78.3	71.4	54.5	60.4

AUC, area under the curve; Sens., sensitivity; Spec., specificity; PPV, positive predictive value; NPV, negative predictive value. These cut-offs are exploratory and are not intended as clinically actionable thresholds; external validation is required.

## 4. Discussion

This study identifies pericoronary adipose tissue thickness as an imaging-derived marker associated with prevalent sarcopenia in hospitalized older adults, whereas no such relationship was observed for epicardial adipose tissue. These findings highlight the potential relevance of regionally distinct cardiac fat depots in reflecting systemic age-related metabolic vulnerability, extending the concept of cardiometabolic imaging beyond traditional cardiovascular risk assessment. Importantly, the persistence of this association after adjustment for age, sex, and BMI suggests that localized adipose tissue characteristics may capture biological processes not adequately represented by generalized measures of adiposity. Because of the retrospective design and non-contemporaneous clinical assessments, our results should be interpreted as associations rather than as a formal diagnostic validation of an imaging biomarker.

The absence of a corresponding association for EAT underscores the biological heterogeneity of cardiac adipose depots. Although both EAT and PCAT contribute to overall cardiac adiposity, their functional roles may differ substantially. Owing to its immediate anatomical proximity to the coronary arterial wall, PCAT is uniquely positioned to reflect localized inflammatory signaling, vascular oxidative stress, and paracrine interactions that extend beyond the hemodynamic influence traditionally attributed to epicardial fat, as demonstrated in imaging studies of perivascular inflammation [22]. In contrast, epicardial adipose tissue has been more consistently linked to systemic cardiometabolic regulation rather than localized vascular inflammatory activity [6]. This region-specific inflammatory milieu may, in turn, contribute to systemic metabolic dysregulation and muscle catabolism, providing a plausible mechanistic framework linking pericoronary adiposity to sarcopenic vulnerability. Importantly, this study was not designed to test mechanistic pathways. We did not measure inflammatory biomarkers, oxidative stress markers, or adipokine profiles, and therefore any mechanistic interpretation should be considered hypothesis-based rather than inferred evidence of causality.

Each 1 mm increase in pericoronary fat thickness was associated with a higher likelihood of sarcopenia in our cohort, suggesting that pericoronary adiposity may serve as a specific imaging marker linked to age-related muscle decline. This observation supports the notion that regionally distributed adipose tissue may convey clinically relevant information beyond generalized measures of adiposity. Moreover, the graded nature of this association implies that PCAT thickness may reflect a continuum of metabolic and inflammatory burden rather than a binary disease state, consistent with contemporary concepts of biological aging and frailty, and the interconnected nature of cardiovascular and musculoskeletal decline in older adults [8]. Given its anatomic proximity to the coronary arterial wall, pericoronary adiposity has been proposed to be linked to local paracrine and inflammatory processes in prior imaging studies. In our cohort, the observed association between PCAT thickness and sarcopenia is consistent with a model in which regionally distributed cardiac adiposity may co-occur with systemic metabolic dysregulation that also relates to muscle decline. Future studies incorporating contemporaneous measures of systemic inflammation, cardiometabolic biomarkers, and CT-based tissue characterization will be required to evaluate whether such biological pathways explain the observed association.

Reinforcing this perspective, the association between PCAT thickness and sarcopenia became more pronounced after adjustment for BMI, indicating that region-specific adipose tissue distribution may convey clinically relevant information beyond overall body mass. This pattern is consistent with a classical statistical suppression effect: because PCAT and BMI are positively correlated (r = 0.34), and higher BMI is associated with lower sarcopenia risk, the unadjusted estimate underestimates the true magnitude of the PCAT–sarcopenia relationship; controlling for BMI removes this masking effect. In an exploratory BMI stratified analysis, the PCAT sarcopenia association appeared numerically larger in participants with BMI ≥ 25 kg/m^2^ (OR 1.48, *p* = 0.008). However, the formal BMI-by-PCAT interaction was not statistically significant (*p* = 0.290); this subgroup observation is hypothesis-generating only and requires confirmation in adequately powered prospective studies. Within the BMI ≥ 25 kg/m^2^ subgroup, higher PCAT thickness was additionally associated with lower skeletal muscle mass and improved discrimination of sarcopenia. These findings suggest that the coexistence of excess adiposity and muscle deterioration may create a metabolic milieu that amplifies adverse remodeling of body composition, in line with the sarcopenic obesity framework, a clinical phenotype characterized by the simultaneous presence of increased fat mass and reduced muscle integrity, which together contribute to heightened cardiometabolic risk beyond the impact of either condition alone [23,24,25]. It should be noted, however, that BMI is an imprecise proxy for adiposity in older adults, as age-related height loss, fluid shifts, and altered body composition reduce its validity as a measure of fat mass. Current ESPEN–EASO guidelines recommend defining sarcopenic obesity using direct body composition measures, such as BIA-derived fat percentage, rather than BMI alone. In the present cohort, BIA-derived fat percentage data were available for 72 of 101 patients; an exploratory analysis stratifying by median fat percentage did not replicate the BMI-stratified pattern, although this secondary analysis was limited by reduced sample size and should be interpreted cautiously. Future studies should use direct adiposity measures to enable more precise phenotyping of the sarcopenic obesity construct.

The ROC analysis indicates that PCAT thickness alone does not provide sufficient accuracy to serve as a stand-alone diagnostic marker for sarcopenia; any incremental value is modest and applies only when PCAT is interpreted alongside conventional clinical variables rather than in isolation. Within the BMI ≥ 25 kg/m^2^ subgroup, an exploratory threshold of 11.44 mm showed high sensitivity and a relatively high negative predictive value; this observation requires external validation and is not intended for clinical use. The optimal threshold was derived using the Youden index, which weights sensitivity and specificity equally; alternative thresholds would shift this balance, but given the modest overall discrimination, none is proposed for clinical screening. When PCAT thickness was evaluated alongside age, sex, and BMI in a combined model, discrimination increased modestly but did not reach statistical significance, indicating that PCAT may contribute incremental information only when integrated with conventional clinical variables and not as a stand-alone measure. PCAT alone yielded an AUC value of 0.637. To formally evaluate the additional discriminative value of PCAT, the AUC of the baseline model including age, sex, and BMI (AUC = 0.667) was compared with that of the PCAT-enhanced model (AUC = 0.754). Although adding PCAT to the baseline model increased the AUC from 0.667 to 0.754, this increase was not statistically significant according to the DeLong test (ΔAUC = 0.087, *p* = 0.083). The likelihood ratio test confirmed that PCAT significantly improved model fit (χ^2^ = 11.68, *p* < 0.001). However, the fact that this significant improvement in model fit did not translate into a statistically significant gain in AUC-level discrimination suggests that, although PCAT statistically contributes to the discrimination equation, its ability to provide clinically meaningful reclassification at the individual patient level remains modest in the current sample size. Accordingly, the incremental discriminative value of PCAT should be interpreted not as a definitive finding, but rather as a supportive signal. The integrated discrimination improvement was 0.104 (95% CI −0.012 to 0.220; *p* = 0.080; bootstrap SE = 0.059, 2000 resamples), indicating a trend toward improved discrimination that did not reach the conventional threshold for statistical significance.

A further observation warrants consideration. In the BMI ≥ 25 kg/m^2^ subgroup, the LOWESS-fitted curve suggested a non-monotonic relationship between PCAT thickness and skeletal muscle mass: muscle mass appeared stable or slightly higher at PCAT values up to approximately 10 mm, then declined progressively beyond this level. This pattern, if confirmed in larger cohorts, could indicate a threshold-dependent effect in which pericoronary adipose tissue exerts negligible or even neutral metabolic influence at lower volumes but transitions to a pro-inflammatory, lipotoxic phenotype at higher thicknesses, a concept consistent with the known dual metabolic role of visceral adipose depots. To formally test for non-linearity, a quadratic term (PCAT^2^) was added to the age- and sex-adjusted linear regression model for skeletal muscle mass within the BMI ≥ 25 kg/m^2^ subgroup; the quadratic coefficient was not statistically significant (*p* = 0.897), and a corresponding likelihood ratio test in the logistic model for sarcopenia likewise did not confirm a non-linear association (*p* = 0.064). These results suggest that the visual inflection in the LOWESS curve more likely reflects sampling variability than a true biological threshold. Because Spearman correlation captures only monotonic associations, these results should be interpreted with the caveat that a non-linear relationship, if present, would be underestimated by a single correlation coefficient. An intriguing finding of the present study was the sex-dependent association between PCAT thickness and skeletal muscle mass. In our cohort, higher PCAT values were associated with lower muscle mass in men, whereas no meaningful association was observed among women. Although the biological basis of this difference remains uncertain, sex-related variations in adipose tissue distribution, hormonal milieu, and inflammatory regulation may partly explain this divergence, as previously demonstrated in studies of sex-specific adipose tissue biology [26]. Importantly, the relatively small sample size necessitates cautious interpretation, and this observation should therefore be regarded as hypothesis-generating rather than conclusive. Larger studies are required to determine whether regional adiposity exerts differential cardiometabolic and musculoskeletal effects across sexes in aging populations. However, the borderline significance of the quadratic term in the logistic model (*p* = 0.064) in a subgroup of only 59 patients suggests that the present analysis may have been underpowered to detect a threshold-dependent effect, and a non-linear relationship cannot be excluded. Larger studies with sufficient power to employ restricted cubic spline models are warranted.

Several methodological considerations should be acknowledged. In the present study, PCAT thickness was quantified as a linear measurement on clinically acquired unenhanced thoracic CT scans. Although this approach is practical and readily applicable in routine clinical settings without dedicated cardiac imaging protocols, it may be susceptible to respiratory motion artifacts, interobserver variability in coronary segment selection, and limited comparability with volumetric or attenuation-based measurements derived from ECG-gated cardiac CT or coronary CT angiography.

Measurements were performed by a single radiologist; although intraobserver reproducibility was excellent (ICC 0.965–0.968), interobserver reproducibility could not be assessed in this retrospective cohort. This is a limitation of the article. PCAT was assessed as a linear thickness on non-ECG gated thoracic CT rather than by volumetric or attenuation-based techniques, which are more susceptible to motion artifact and segment-selection variability. A standardised protocol with thin-slice reconstruction, multi-territory averaging, and exclusion of oblique sections was used to mitigate these sources of error; nevertheless, prospective studies with standardised, ideally ECG gated acquisition and full intra and interobserver reproducibility testing are warranted.

Interobserver reliability could not be assessed in this study, which is a limitation of our study.

Linear, cross-sectional measurements cannot fully capture physiological anatomical variation such as the intramyocardial course of coronary arteries or the focal, heterogeneous absence of epicardial fat which may reduce measurement reliability; this remains an inherent limitation of the 2D approach.

Data regarding the timing of previous myocardial infarction, and especially recent acute coronary syndrome, were not consistently available. Since post-infarction inflammatory and fibrotic remodeling can alter epicardial and pericoronary tissue characteristics, unmeasured MI history and timing may contribute to residual confounding in the observed relationships.

Nonetheless, the use of standard thoracic CT reflects real-world clinical practice and highlights the feasibility of opportunistic cardiac fat assessment in hospitalized older adults [27,28].

Inclusion allowed up to 90 days between thoracic CT and BIA, and in hospitalized older adults body composition can change materially over that interval through acute illness, immobilization, nutritional fluctuation, or deconditioning. The median CT–BIA interval in this cohort was 36 days (IQR 10–81), and the interval did not differ by sarcopenia status. Sensitivity analyses restricted to ≤60 days (n = 58) and ≤30 days (n = 46) preserved the PCAT–sarcopenia association with materially similar effect estimates (adjusted OR 1.48 and 1.52 respectively, both *p* < 0.05), arguing against attenuation by temporal misclassification. Nevertheless, prospective designs with simultaneous imaging and body-composition assessment would be required to fully eliminate this concern.

Skeletal muscle mass was estimated using BIA, which is practical and widely used but can be sensitive to hydration status. In hospitalized older adults, acute illness, diuretic therapy, fluid shifts, and subclinical edema may affect impedance-derived estimates and could lead to misclassification of muscle mass and sarcopenia status. Although we attempted to standardize measurements and excluded patients with severe edema or major electrolyte imbalance, residual measurement error related to hydration cannot be fully excluded.

Although tissue sampling was not performed in our study, previous histological and immunohistochemical studies support biological heterogeneity between cardiac and perivascular fat deposits [29]. However, these mechanisms were not directly evaluated in our cohort. To validate the cellular mechanisms underlying the observed associations, future prospective studies integrating CT-based measurements with circulating and tissue-level histological and immunohistochemical markers are needed. These results demonstrate the feasibility of assessing pericoronary adipose tissue using linear measurements obtained from routinely performed unenhanced thoracic CT examinations in hospitalized older adults. Although this approach does not replace dedicated cardiac imaging protocols, it suggests that the pericoronary fat compartment may convey cardiometabolic and inflammatory information not adequately reflected by global epicardial fat thickness. In this context, region-specific cardiac adiposity may represent an integrative imaging marker of age-related metabolic vulnerability. While further prospective validation is required, the present findings support the potential role of opportunistic imaging biomarkers in improving multidimensional geriatric risk evaluation and in advancing understanding of the interplay between cardiovascular and musculoskeletal aging.

## 5. Conclusions

In hospitalized older adults, PCAT thickness measured on routine non-contrast thoracic CT was associated with prevalent sarcopenia after adjustment for age, sex, and BMI, whereas EAT thickness was not. These data underscore the heterogeneity of cardiac adiposity and suggest that the pericoronary fat compartment may be more informative for skeletal muscle status than the broader epicardial fat layer. In exploratory analyses, the association was numerically larger in participants with BMI ≥ 25 kg/m^2^; this BMI-stratified observation is hypothesis-generating only and requires confirmation in adequately powered prospective studies before any clinical application can be considered. Nonetheless, the formal interaction test was not statistically significant, and these subgroup findings require confirmation in larger prospective cohorts with standardized sarcopenia ascertainment and direct measures of adiposity.

## Data Availability

Data supporting the findings of this study can be obtained but there are restrictions on the availability of these data, these data were used under license for the current study and are therefore not publicly available. However, data can be obtained from the corresponding author upon reasonable request.

## Supplementary Materials

The following supporting information can be downloaded at: https://www.mdpi.com/article/10.3390/medicina62061115/s1.

## Abbreviations

EAT	epicardial adipose tissue
PCAT	pericoronary adipose tissue
CT	computed tomography
BMI	body mass index
BIA	bioelectrical impedance analysis
ADL	Activities of Daily Living Scale
IADL	Instrumental Activities of Daily Living Scale
GDS	Geriatric Depression Scale
MNA-SF	short form of the Mini Nutritional Assessment
MMSE	Mini-Mental State Examination
TKK	A Takei 5101 digital dynamometer
EPV	events-per-variable
