# Supplementary material for: Pericoronary, but Not Epicardial, Cardiac Fat Thickness Is Associated with Sarcopenia in Hospitalized Older Adults
Source: Medicina (Kaunas). 2026 Jun 8;62(6):1115. doi: 10.3390/medicina62061115 (PMC13302967; doi:10.3390/medicina62061115)
Supplement: Supplementary file 1 [file medicina-62-01115-s001.zip › medicina-4267565-supplementary.pdf]

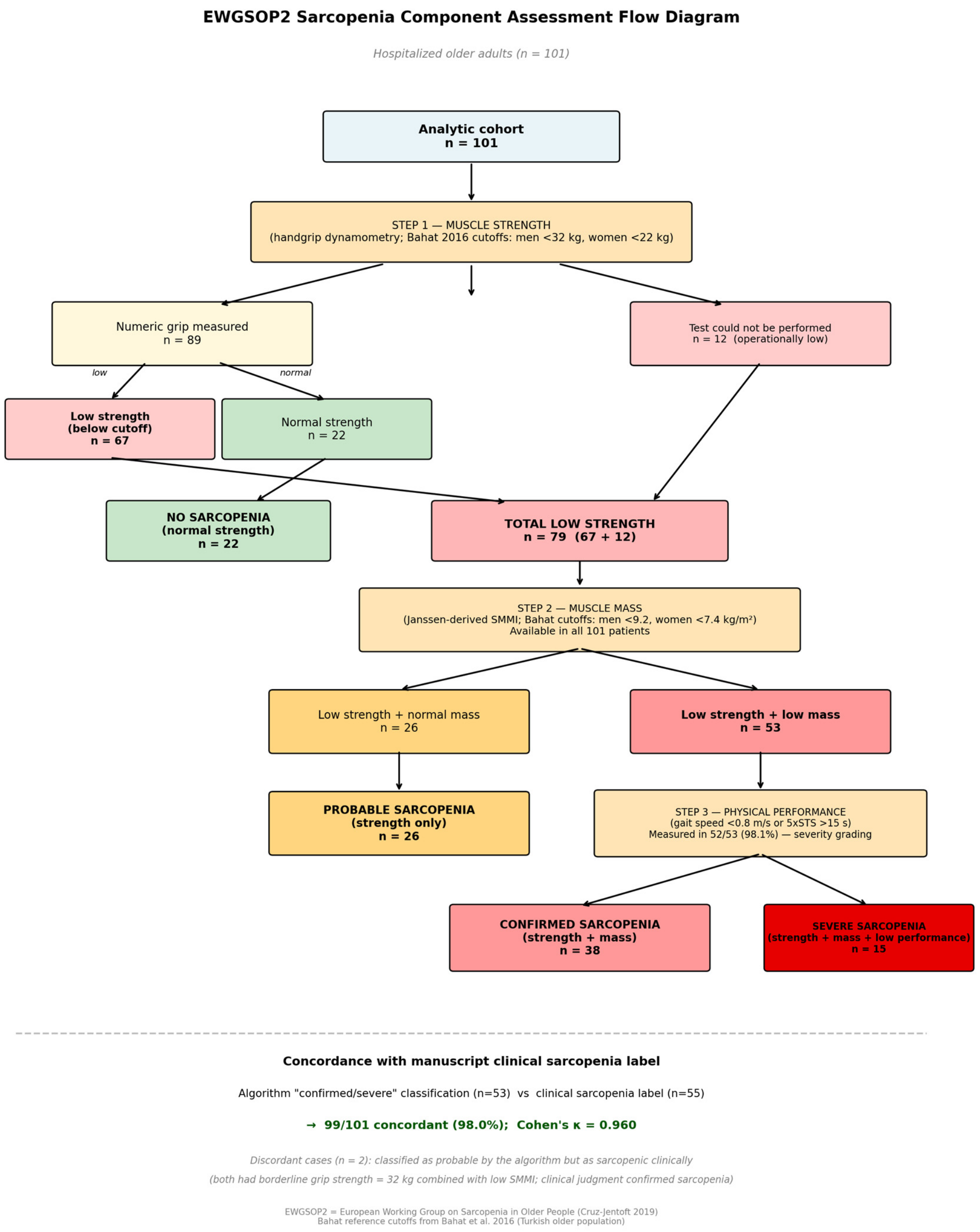

Supplementary Figure S1. EWGSOP2 sarcopenia component assessment flow diagram for the analytic cohort (n=101). Step 1 (muscle strength by handgrip dynamometry, Bahat 2016 cutoffs), Step 2 (muscle mass by Janssen-derived SMMI), and Step 3 (physical performance for severity grading) are shown with patient counts at each branch. Numeric handgrip values were available in 89 patients; 12 without a measurable grip were operationally classified as having low strength. An internal consistency audit re-applying the formal algorithm reproduced the recorded clinical sarcopenia label in 99 of 101 patients (98.0%; Cohen's κ = 0.960).

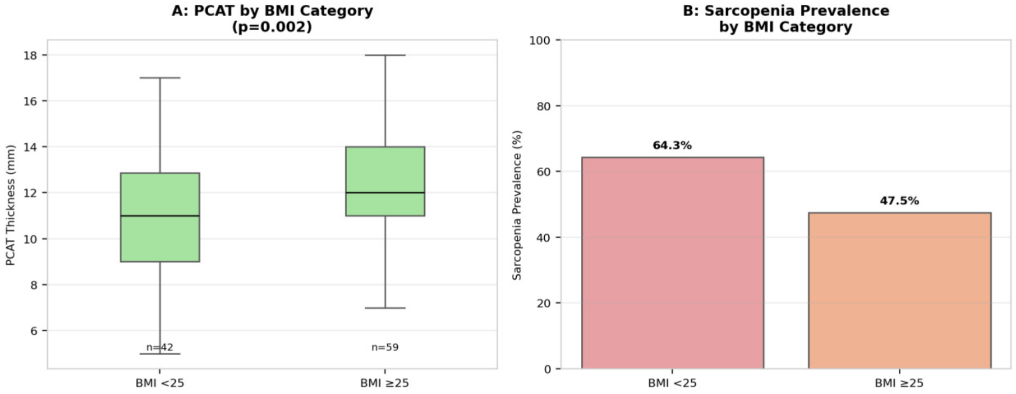

Supplementary Figure S2. Results stratified by BMI category. (A) PCAT thickness was greater in the BMI ≥25 kg/m² group (Student's t-test, p=0.002). (B) Sarcopenia was more prevalent among those with BMI <25 kg/m² (64.3% vs 47.5%).

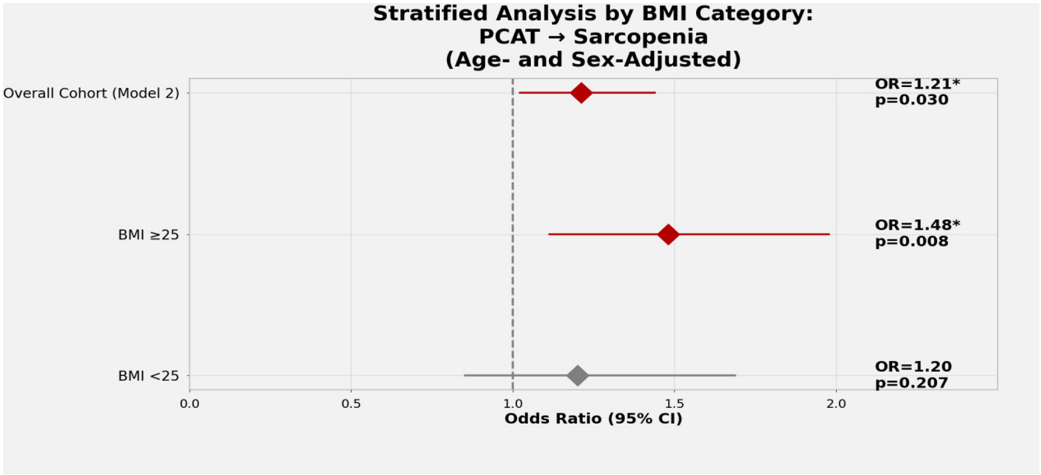

Supplementary Figure S3. PCAT–sarcopenia association by BMI stratum in age- and sex-adjusted logistic regression. A significant association was found in the BMI ≥25 kg/m² group (OR=1.48, 95% CI 1.11–1.98; p=0.008) but not in the BMI <25 kg/m² group (OR=1.20, 95% CI 0.90–1.60; p=0.207).

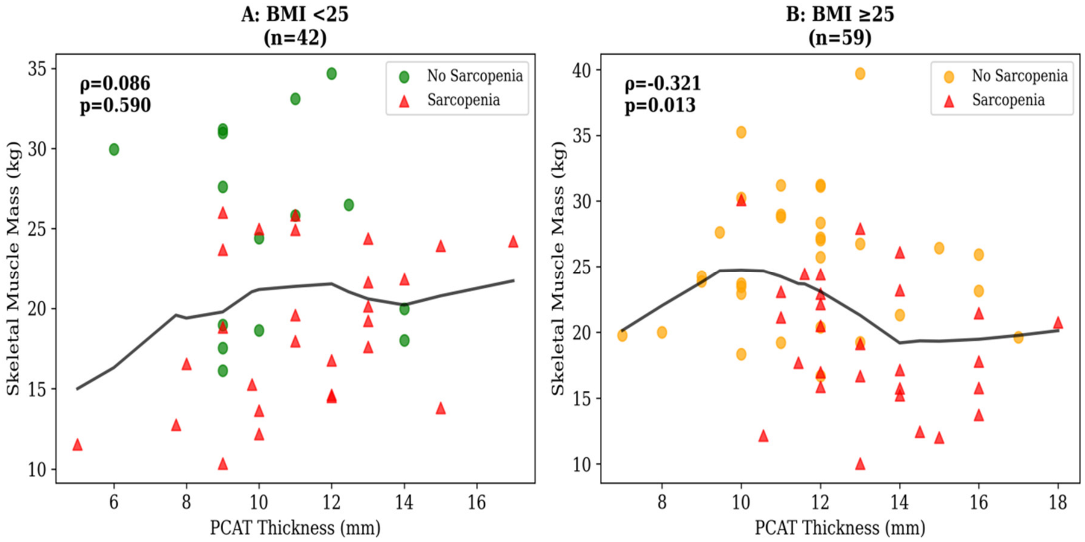

Supplementary Figure S4. PCAT thickness versus skeletal muscle mass by BMI category. (A) BMI <25 kg/m²: no significant correlation (Spearman's ϱ = 0.086, p=0.590). (B) BMI ≥25 kg/m²: significant negative correlation (Spearman's ϱ = −0.321, p=0.013). Red triangles mark sarcopenic participants. LOWESS curves are fitted to visualize trends.

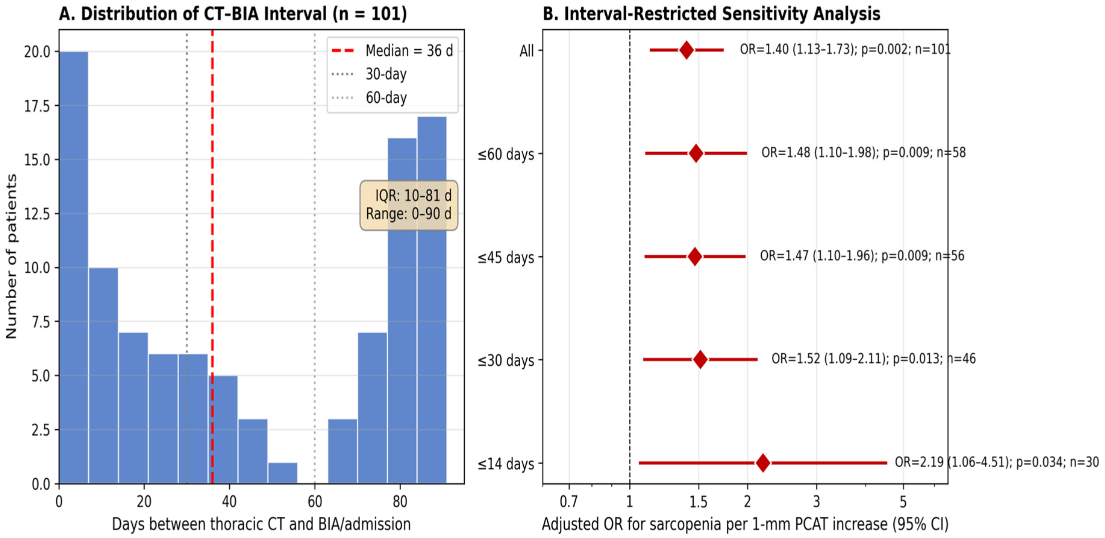

Supplementary Figure S5. Distribution of the interval between thoracic CT and bioelectrical impedance analysis (BIA), and interval-restricted sensitivity analysis of the PCAT–sarcopenia association. (A) Histogram of the CT–BIA interval (median 36 days, IQR 10–81 days; range 0–90 days). (B) Forest plot of the adjusted PCAT odds ratio (age, sex, BMI) in the full cohort and in subsets restricted to ≤60 and ≤30 days, showing that the association was preserved and numerically larger at shorter intervals.
